# Effect of emphysema on AI software and human reader performance in lung nodule detection from low-dose chest CT

**DOI:** 10.1186/s41747-024-00459-9

**Published:** 2024-05-20

**Authors:** Nikos Sourlos, GertJan Pelgrim, Hendrik Joost Wisselink, Xiaofei Yang, Gonda de Jonge, Mieneke Rook, Mathias Prokop, Grigory Sidorenkov, Marcel van Tuinen, Rozemarijn Vliegenthart, Peter M. A. van Ooijen

**Affiliations:** 1 Department of Radiology, University Medical Center of Groningen, Groningen, 9713GZ The Netherlands; 2 Department of Oral Surgery of the Medical Spectrum Twente (MST), Enschede, 7500KA The Netherlands; 3 DataScience Center in Health (DASH), University Medical Center Groningen, Groningen, 9713GZ The Netherlands; 4 Department of Epidemiology, University Medical Center Groningen, Groningen, 9713GZ The Netherlands; 5 Department of Radiology, Martini Hospital, Groningen, 9728NT The Netherlands; 6 Department of Radiation Oncology, University Medical Center Groningen, Groningen, 9713GZ The Netherlands

**Keywords:** Artificial intelligence, Emphysema, Multiple pulmonary nodules, Software validation, Tomography (x-ray computed)

## Abstract

**Background:**

Emphysema influences the appearance of lung tissue in computed tomography (CT). We evaluated whether this affects lung nodule detection by artificial intelligence (AI) and human readers (HR).

**Methods:**

Individuals were selected from the “Lifelines” cohort who had undergone low-dose chest CT. Nodules in individuals without emphysema were matched to similar-sized nodules in individuals with at least moderate emphysema. AI results for nodular findings of 30–100 mm^3^ and 101–300 mm^3^ were compared to those of HR; two expert radiologists blindly reviewed discrepancies. Sensitivity and false positives (FPs)/scan were compared for emphysema and non-emphysema groups.

**Results:**

Thirty-nine participants with and 82 without emphysema were included (*n* = 121, aged 61 ± 8 years (mean ± standard deviation), 58/121 males (47.9%)). AI and HR detected 196 and 206 nodular findings, respectively, yielding 109 concordant nodules and 184 discrepancies, including 118 true nodules. For AI, sensitivity was 0.68 (95% confidence interval 0.57–0.77) in emphysema *versus* 0.71 (0.62–0.78) in non-emphysema, with FPs/scan 0.51 and 0.22, respectively (*p* = 0.028). For HR, sensitivity was 0.76 (0.65–0.84) and 0.80 (0.72–0.86), with FPs/scan of 0.15 and 0.27 (*p* = 0.230). Overall sensitivity was slightly higher for HR than for AI, but this difference disappeared after the exclusion of benign lymph nodes. FPs/scan were higher for AI in emphysema than in non-emphysema (*p* = 0.028), while FPs/scan for HR were higher than AI for 30–100 mm^3^ nodules in non-emphysema (*p* = 0.009).

**Conclusions:**

AI resulted in more FPs/scan in emphysema compared to non-emphysema, a difference not observed for HR.

**Relevance statement:**

In the creation of a benchmark dataset to validate AI software for lung nodule detection, the inclusion of emphysema cases is important due to the additional number of FPs.

**Key points:**

• The sensitivity of nodule detection by AI was similar in emphysema and non-emphysema.

• AI had more FPs/scan in emphysema compared to non-emphysema.

• Sensitivity and FPs/scan by the human reader were comparable for emphysema and non-emphysema.

• Emphysema and non-emphysema representation in benchmark dataset is important for validating AI.

**Graphical Abstract:**

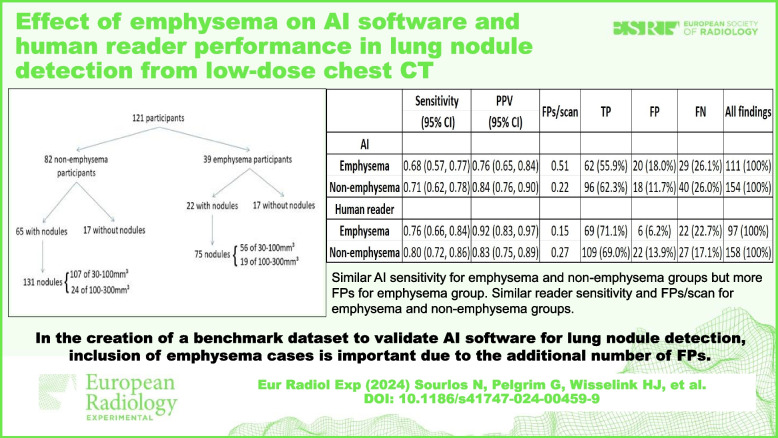

## Background

Lung cancer is one of the deadliest cancers [1]. Lung nodules detected on computed tomography (CT) examinations may be an early sign of lung cancer. Therefore, it is of great importance to detect these nodules on CT, even if most of these turn out to be benign [2]. Lung nodule detection is a time-consuming task for radiologists. Many, artificial intelligence (AI)-based, automated lung nodule detection algorithms have been developed aiming to help radiologists in their daily clinical routine or in screening setting. Most of these algorithms are based on the publicly available LUNA16 dataset [3]. However, it is unclear if these algorithms perform well in different settings as they may contain biases that limit generalizability [4]. Because of these biases, the AI algorithms may not be accurate for use in all cohorts and should be used only in similar populations to the ones on which these algorithms were developed and validated, unless adequate performance is confirmed in the target population [4].

The performance of different AI solutions for lung nodule detection was previously compared to that of human readers [5–7]. The sensitivity of AI solutions developed based on the LUNA16 dataset ranged from 0.79 to 0.98 and the false positives (FPs)/scan from 1 to 8 [7]. For commercial software, the sensitivity is usually higher than that of the radiologist but commonly at the expense of more FPs/scan. For example, Chamberlin et al. [8] showed that the sensitivity of AI lung nodule detection software was 1.00 and specificity moderate (0.71), with good agreement between AI and expert radiologists (Cohen’s *κ* = 0.74). So far, there is no evidence of the detection performance of AI software in subcohorts, such as individuals with emphysema.

Emphysema, a parenchymal lung disease often related to smoking, changes the overall appearance of the lung on CT images and is associated with a higher prevalence of lung nodules and scars [9, 10]. In low-dose chest CT screening studies, emphysema was present in over 20% of participants screened for lung cancer [11]. Due to architectural distortion and the presence of scars in emphysema, AI or a human reader may miss nodules but could also find more FPs. Because scars may be interpreted as nodules, irregularly shaped nodular findings, which could be cancer-mimicking postinflammatory scars, may complicate the correct identification of nodules [12]. Furthermore, larger nodules exist in cases with emphysema [10], and in areas with severe emphysema, lung cancer tends to develop more often [13, 14]. It is therefore crucial to correctly detect nodules in individuals with emphysema and particularly in emphysematous regions, a task that an automated algorithm could help to accomplish, albeit at the possible expense of additional FPs [15].

The goal of this study is to evaluate whether sensitivity or FPs/scan in AI lung nodule detection software differs between scans of individuals with and without emphysema, and to compare these results to those of a human reader, using a consensus expert panel review. The motivation for conducting this study stems from the uncertainty surrounding potential biases within AI software and its ability to generalize to diverse populations beyond its original training set. For this purpose, we used a commercially available software product as a proof of principle for studying emphysema as a potential source of bias in AI performance.

## Methods

### Study population and CT acquisition

The dataset used in this study is part of the ImaLife (Imaging in Lifelines) cohort [16], a subcohort of Lifelines with participants aged 45 years and above who underwent low-dose chest CT scanning between 2017 and 2022. Lifelines is a multidisciplinary prospective population-based cohort study examining in a unique three-generation design the health and health-related behaviors of 167,729 persons living in the North of the Netherlands. It employs a broad range of investigative procedures in assessing the biomedical, socio-demographic, behavioral, physical, and psychological factors that contribute to the health and disease of the general population, with a special focus on multi-morbidity and complex genetics. Initiated in 2006, Lifelines conducts repeated follow-up rounds, where participants complete additional questionnaires, provide biosamples, and undergo physical examinations.

ImaLife participants underwent a noncontrast chest CT scan using a third-generation dual-source CT (SOMATOM Force, Siemens Healthineers, Erlangen, Germany), with a low-dose protocol. The effective dose for the scans was between 0.6 and 1.8 mSv. CT acquisition parameters can be found in a previous report [16]. Information about demographics was available from the Lifelines database [17]. Informed consent was obtained from all participants prior to participating in the ImaLife study and the medical ethics committee of the University Medical Center of Groningen approved the study.

For this study, we selected individuals with at least moderate emphysema, as well as a comparison group without emphysema. These groups are described in more detail below.

### Scan evaluation and subcohort selection

At the initial reading of the ImaLife CT scans, lung nodules were detected and quantified by a trained technical medicine graduate (G.P.) with 3 years of experience under a radiologist’s supervision (M.R. with 6 years of experience reading chest CTs), using dedicated software (Pulmo3D, Syngo.via VB40, Siemens Healthineers). A smooth kernel Br40 was used for lung nodule detection and a hard kernel (Qr59) for nodule quantification. The reader registered a maximum of ten nodules per scan. If a participant had more than ten nodules, the ten largest were registered. Nodules were classified into size categories based on volume: 30–100 mm^3^, 101–300 mm^3^, and > 300 mm^3^. Nodules smaller than 30 mm^3^ were not characterized. For the current study, nodules larger than 300 mm^3^ were disregarded since only a few cases were available, and detection is relatively easy for larger-sized nodules. The presence of at least trace emphysema was noted at the initial reading; all other individuals were noted as having no emphysema.

For 1,533 scans available in the ImaLife database, detailed emphysema classification was performed by a trained technical physician with 3 years of experience (H.J.W.) or a radiologist with 5 years of experience (X.Y.). Evaluations were performed with an extended, lobar-based version of emphysema classification [18, 19]. The lobe with the most severe emphysema classification was considered for the overall emphysema classification for that participant, resulting in a classification according to Fleischner [19]. Cases without emphysema presence were also noted. For the current study, all cases from those 1,533 with at least moderate emphysema (moderate, confluent centrilobular, or advanced destructive emphysema) were selected. Cases with trace and mild emphysema were excluded from our analysis.

To permit valid comparison between emphysema and non-emphysema cases, in view of the expected larger size of nodules in emphysema cases, we matched each nodule in emphysema cases to a similar sized nodule (± 10 mm^3^) in ImaLife individuals without emphysema. During the matching process, it was ensured that a nodule from the same volume subgroup was selected. For example, for a nodule sized 98 mm^3^ in an emphysema participant, we searched the database for a nodule in a non-emphysema participant with nodule size from 88 mm^3^ (as max 10 mm^3^ lower) to 100 mm^3^ (upper limit of size category 30–100 mm^3^). The maximum number of nodules per emphysema participant was ten (the ten largest were noted). In the case of multiple nodules in emphysema participants, each individual nodule was size-matched with a nodule of similar size among the large group of non-emphysema participants. Thus, for multiple nodules in an emphysema participant, matching could consist of nodules in different non-emphysema participants. Because non-emphysema participants with a size-matched nodule could have additional, non-matched nodules (radiologists may have found more than 1 nodule in that participant), the non-emphysema group contained more nodules. We randomly selected participants in the non-nodule, non-emphysema group to match the number of participants without nodules in the emphysema group.

### AI nodule detection

Scans of included participants were sent to the AI-Rad Companion chest CT research application (Version 08/2022, Siemens Healthineers). According to vendor recommendations, reconstruction kernel Qr59 was used. After processing, the AI tool returned the original scan overlayed with red boxes around the findings it considered as nodules. Similar to the human reader, the AI algorithm only registered a maximum of 10 nodules per scan by selecting the 10 largest nodules from the nodule candidates detected.

### Evaluation of nodule detection discrepancies and nodule volume

Nodular findings detected by both AI and the human reader were considered true-positive nodules (TPs). Discrepancies between findings for AI and the human reader were listed. Two experienced radiologists (R.V. with 17 and G.J. with 13 years of experience in chest CT) blinded to the origin of the finding (AI or human reader) in consensus decided if discrepancies were true nodules or not. Furthermore, they added a confidence score (from 0, random guess to 5, extremely confident) and provided information on the type of finding (*e.g.*, type of non-nodular finding, nodule, or lymph node). Typical perifissural nodules (PFNs) and bronchovascular lymph nodes were included in our main analysis. These findings are non-malignant [20, 21] and do not need follow-up if their size is below 524 mm^3^ [22]. Therefore, they can be considered less clinically relevant. In secondary statistical analyses, we excluded typical PFNs and bronchovascular lymph nodes.

Nodule size was based on the volume of the solid component of the nodule (if > 30 mm^3^) (either whole in case of solid nodule, or part in case of part-solid nodule) and on the non-solid volume in case of non-solid nodule or a part-solid nodule with solid part less than 30 mm^3^. For findings only detected by AI, volume size was retrospectively measured with syngo.via software (Version VB60, Siemens Healthineers), to establish a common reference standard for AI and reader: that volume was then used to assign the finding into the appropriate volume size subgroup. For consistency, nodules smaller than 30 mm^3^ or larger than 300 mm^3^ detected only by AI were not included in our analysis. Findings found only by AI as < 30 mm^3^ but manually measured within syngo.via as > 30 mm^3^ were also excluded from analysis (*n* < 5).

### Statistical analysis

After the consensus was read by the radiologists, sensitivity, positive predictive value (PPV), FPs/scan, TPs, FPs, and false negatives (FNs) were determined. We used the definition of true negatives (TNs) as proposed by Chamberlin et al. [8] who defined TNs as cases in which no nodules were reported by the initial human reading and no findings were found by AI. The primary analysis was performed by including lymph nodes. Subsequently, secondary analyses were performed after the exclusion of lymph nodes and, for the whole dataset, separately for the two size categories. To estimate the variability in metric values, 95% confidence intervals (CIs) were calculated for sensitivity, and PPV, using Wilson’s method for proportions with continuity correction [23]. To investigate if a difference exists in sensitivity between emphysema and non-emphysema groups for AI and for the reader, as well as between AI and reader for each of the emphysema/non-emphysema groups, the overlap of CIs was utilized as a metric. If the ranges of two CIs did not overlap, we interpreted that as a significant difference. In addition, *χ*^2^ tests were performed to evaluate whether TP rate (observed TP/total TP), as a proxy of sensitivity, differed significantly between AI and reader for both emphysema and non-emphysema. A McNemar test was not deemed appropriate for this purpose because the comparison of two independent detection methods against the consensus panel each yields separate FN and FP counts, which cannot be combined in a 2 × 2 matrix. The use of a human reader as a standard against which to compare AI performance also was considered suboptimal because of the focus of this investigation on the true performance of AI in nodule detection. Similarly, a formal statistical comparison of AI *versus* consensus panel and human reader *versus* consensus read was not practical due to the absence of TNs for the consensus panel. For comparing the number of FPs/scan, Mann–Whitney *U* testing was utilized for the comparison of AI between emphysema and non-emphysema groups (same for human reader), as well as for the comparison of AI *versus* reader between emphysema and non-emphysema groups. For categorical variables (sex, ever smoker) in the demographics table, the *χ*^2^ test was used. To check for the difference in means between the emphysema and non-emphysema groups for numeric variables, the Mann–Whitney* U* test was utilized since in a few cases the distribution of data stratified by emphysema condition was not normal (checked with the Shapiro–Wilk test). A *p*-value smaller than 0.05 was considered statistically significant. The statistical analysis was performed in Python using the SciPy statistical library [24].

## Results

### Participant selection and characteristics

After the exclusion of trace and mild emphysema participants (*n* = 608) and cases with at least moderate emphysema but without information about the presence of nodules (*n* = 4), this study included 39 emphysema participants with at least moderate emphysema (12 with advanced destructive/confluent emphysema and 27 with moderate emphysema). After matching for nodule size, 82 non-emphysema participants were included. These were selected from the 882 individuals without emphysema. The total sample size of 121 included therefore 32.2% participants with emphysema and 67.8% non-emphysema cases. The characteristics of participants of the emphysema/non-emphysema groups are shown in Table 1. The age of the participants was 60.6 ± 8.3 years (mean ± standard deviation), 58/121 (47.9%) were male, and 88/121 (72.7%) were ever smokers. Individuals with emphysema comprised more ever smokers and had more smoking pack years compared to individuals without emphysema.

**Table 1:** Participant characteristics for the emphysema/non-emphysema groups

All participants (*n* = 121)	Participants with emphysema (*n* = 39)^a^	Participants without emphysema (*n* = 82)	*p*-value
58 (47.9%)	23 (59.0%)	35 (42.7%)	0.140^b^
60.6 ± 8.3	62.7 ± 8.0	59.5 ± 8.3	0.053^c^
81.8 ± 14.2	78.5 ± 12.7	83.3 ± 14.6	0.144^c^
174.7 ± 9.8	176.3 ± 9.4	173.9 ± 9.8	0.199^c^
88 (72.7%)	35 (89.7%)	53 (64.6%)	**0.010**^b^
17.3 ± 16.5	28.9 ± 16.1	9.9 ± 11.7	** < 0.001**^c^

Missing values: smoking status *n* < 10, pack years *n* = 35

^a^27 individuals (69.2%) showed moderate emphysema, and 12 (30.8%) individuals showed advanced/confluent emphysema. Bold values denote a significant difference (*p* < 0.050)

^b^Comparison of the proportion of males or ever smokers between emphysema and non-emphysema

^c^Comparison between mean values of a given continuous attribute between emphysema and non-emphysema groups﻿

**Fig. 1:**
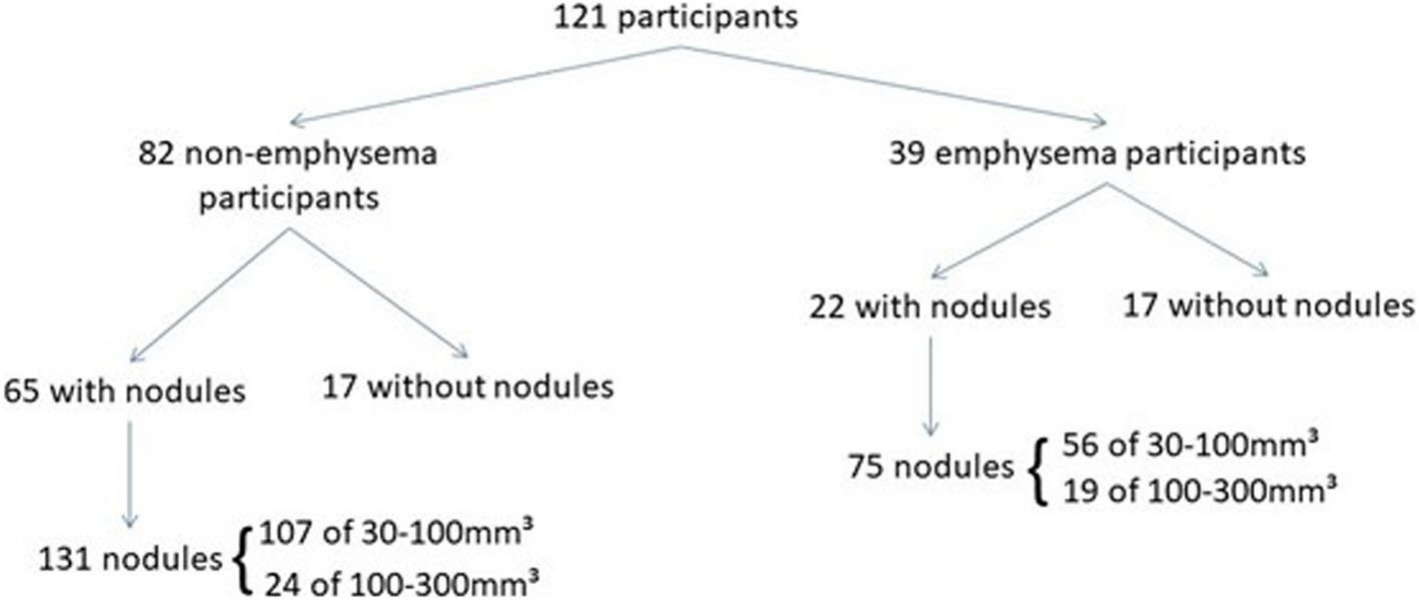
Flowchart depicting participant selection process. Note that because matching was guaranteed for nodules from emphysema patients, and because some participants had more than one nodule, this procedure yielded more nodules in the non-emphysema participants

Seventy-five nodules with a volume of 30–300 mm^3^ were found based on initial reading in individuals with emphysema. Of these, 73 nodules (97.3%) could be size-matched to a nodule in 65 participants without emphysema. In some individuals, more than one nodule could be size-matched. Additional (non-matched) nodules were found in the participants without emphysema, resulting in a total of 131 nodules. In total, nine subsolid nodules (three part-solid, six non-solid) were included. Seventeen participants in the emphysema group lacked nodules, matched to 17 individuals without nodules in the non-emphysema group. A flowchart of the selection process can be seen in Fig. 1.

**Fig. 2:**
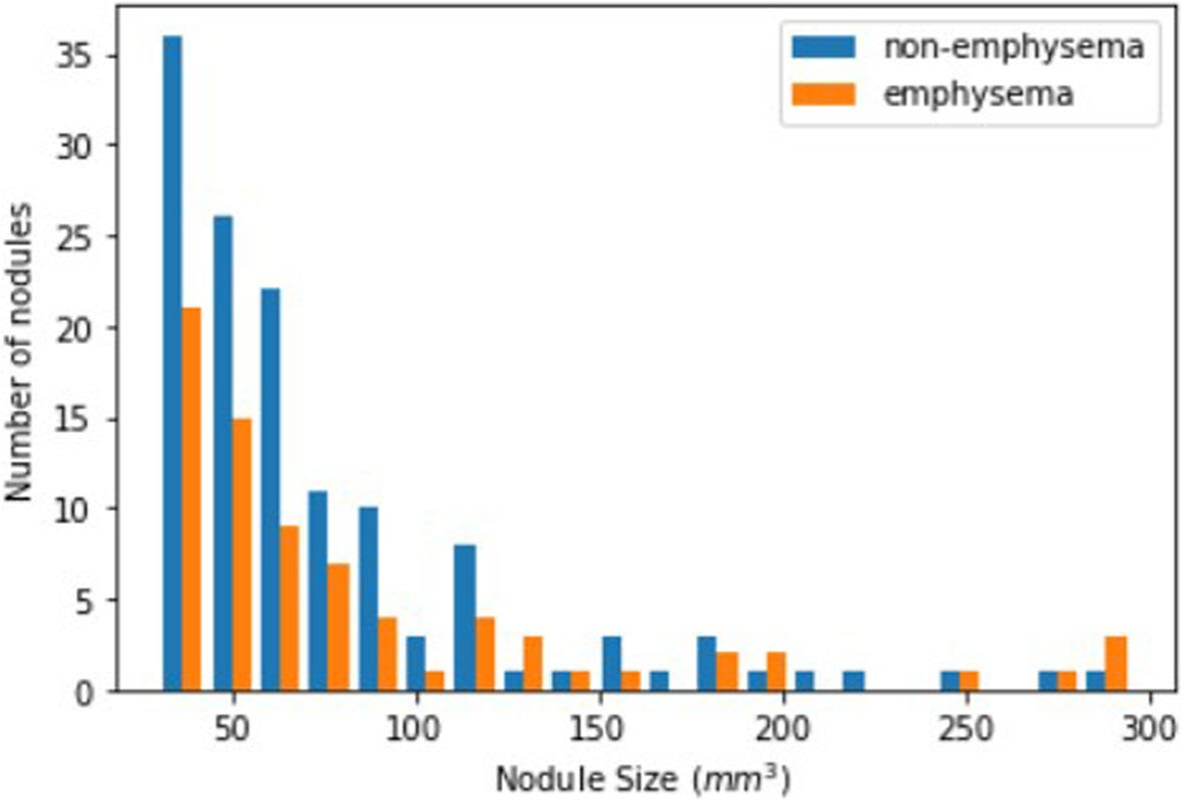
Distribution of the number of nodules based on nodule size for emphysema and non-emphysema groups after matching, for findings detected on the initial reading. More nodules were included from the non-emphysema group due to the nature of the matching procedure

The distribution of nodule sizes in the emphysema and non-emphysema groups is shown in Fig. 2. Both groups showed similar nodule size and relative size distribution: emphysema group, median 58.0 mm^3^, interquartile range 56.5 *versus* non-emphysema group, median 59.0 mm^3^, interquartile range 43.5 mm^3^ (*p* = 0.930).

### Analysis of discrepancies in nodular findings

AI detected in total 196 nodular findings and the human reader 206. Overall, 109 (concordant) nodules were detected by both AI and human reader; 73 of 30–100 mm^3^ nodules (24 in emphysema) and 36 of 101–300 mm^3^ nodules (16 in emphysema). Four cases had at least 10 nodules detected during the initial reading (AI also found at least 10 nodules), while there were 2 cases in which AI detected 10 nodules, but the human reader fewer than 10.

There were 184 discrepant findings, *i.e*., findings found by either AI or human reader: 136 sized 30–100 mm^3^ (52 in emphysema) and 48 of 101–300 mm^3^ (25 in emphysema). Of the 184 discrepancies, 118 were classified as true lung nodules (64.1%) by the consensus panel. Of these 118 true nodules, 49 were detected by AI alone (22 in emphysema) and 69 by the human reader only (29 in emphysema). Most of these additional true nodules were PFNs or bronchovascular lymph nodes (96/118, 81.4%). One finding measured by AI as < 30 mm^3^ but in syngo.via as > 30 mm^3^ was excluded from our analysis. For three findings in the emphysema group and twelve findings in the non-emphysema group, the consensus panel was less than confident on the designation of the nodular finding (confidence score ≤ 3).

### Performance evaluation and comparison

AI’s sensitivity was similar for the emphysema (0.68 (95% CI 0.57–0.77)) and non-emphysema (0.71 (0.62–0.78)) groups. A higher number of FPs/scan by the AI in the emphysema group compared to the non-emphysema group was observed (0.51 FPs/scan *versus* 0.22 FPs/scan, *p* = 0.028) (Table 2). The sensitivity of the human reader was similar for the emphysema and non-emphysema groups (0.76 (95% CI 0.66–0.84) *versus* 0.80 (0.72–0.86)). Likewise, the reader had a similar number of FPs in the emphysema and non-emphysema groups (0.15 FPs/scan *versus* 0.27 FPs/scan, respectively, *p* = 0.230). The 95% CI for the sensitivity of AI and the human reader overlapped to a certain extent, for both the emphysema and non-emphysema groups. The sensitivity of AI was not significantly different for either the emphysema (*p* = 0.320) or the non-emphysema group (*p* = 0.090). However, AI had significantly higher FPs/scan than the reader in the emphysema group (*p* = 0.020).

**Table 2:** Comparison of performance of artificial intelligence (AI) and human reader (HR) for emphysema and non-emphysema groups at nodule level

	Sensitivity (95% CI)	PPV (95% CI)	FPs/scan	TPs	FPs	FNs	All findings
AI							
Emphysema	0.68 (0.57, 0.77)	0.76 (0.65, 0.84)	0.51	62 (55.9%)	20 (18.0%)	29 (26.1%)	111 (100%)
Non-emphysema	0.71 (0.62, 0.78)	0.84 (0.76, 0.90)	0.22	96 (62.3%)	18 (11.7%)	40 (26.0%)	154 (100%)
HR							
Emphysema	0.76 (0.66, 0.84)	0.92 (0.83, 0.97)	0.15	69 (71.1%)	6 (6.2%)	22 (22.7%)	97 (100%)
Non-emphysema	0.80 (0.72, 0.86)	0.83 (0.75, 0.89)	0.27	109 (69.0%)	22 (13.9%)	27 (17.1%)	158 (100%)

*CI* Confidence interval, *PPV* Positive predictive value, *FPs/scan* False positives per scan, *TPs* True positives, *FPs* False positives, *FNs* False negatives

**Fig. 3:**
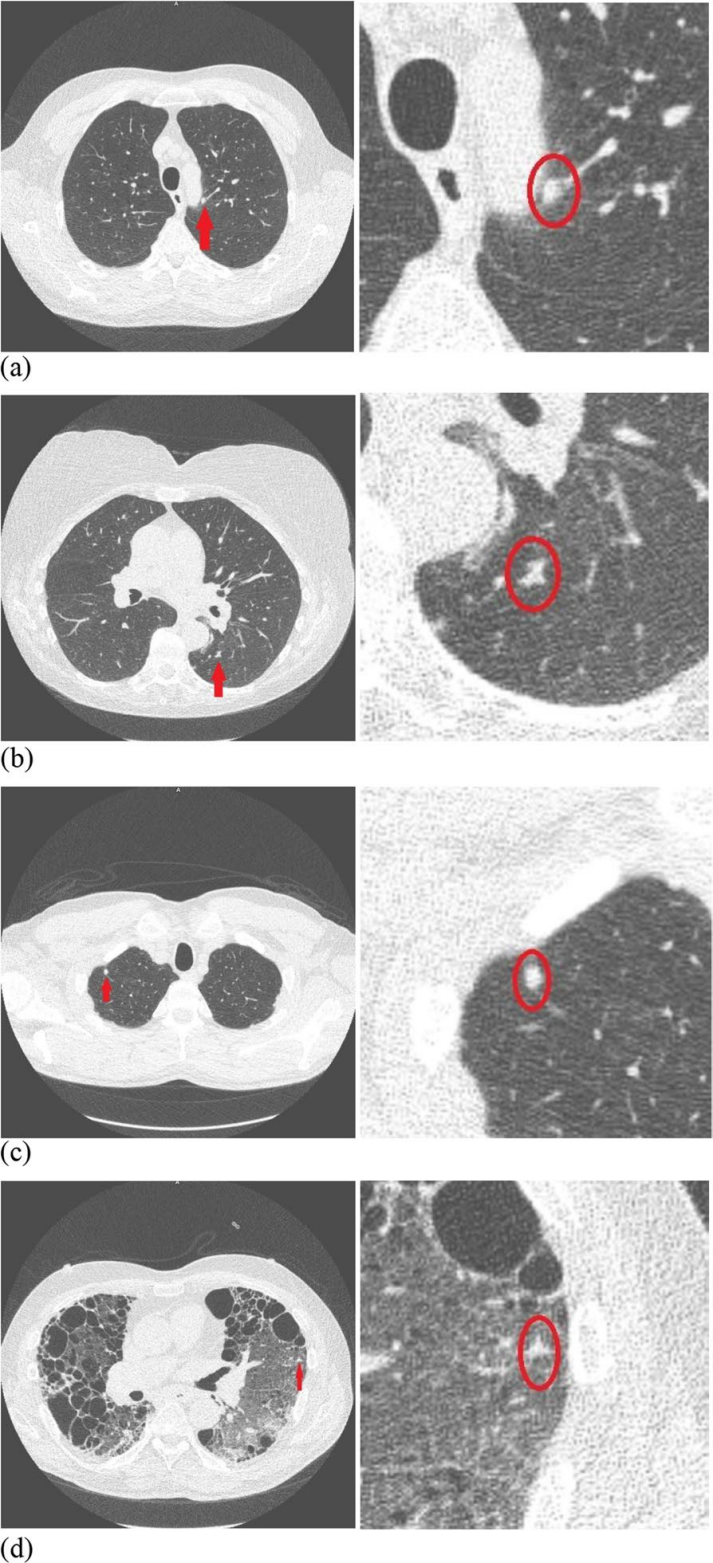
Examples of discrepant nodular findings for artificial intelligence (AI) and human reader (HR): (**a**) true-positive finding detected by AI only (192 mm^3^), (**b**) true-positive finding detected only by HR (154 mm^3^), (**c**) false-positive finding by AI (90 mm^3^), (**d**) false-positive finding by HR (56 mm^3^). On the left side of each figure part is the cross-sectional CT image with the finding highlighted with an arrow, and on the right side is a magnified image

In the emphysema group, most FPs were found for AI (20/26, 76.9% of all FPs of both AI and human reader) and comprised fibrosis/scars (13/26, 50.0% of AI FPs). In the non-emphysema group, most FPs originated from the reader (22/40, 55% of all FP). “Other non-nodules” could represent bone, mucus in bronchi, arthrosis, vessel, consolidation, infection, fat, or atelectasis. Figure 3 illustrates discrepant findings of true nodules and examples of FPs found by only AI or human reader.

A secondary analysis was performed after excluding (benign) typical and bronchovascular lymph nodes from the nodule group (Table 3). This analysis showed a tendency of higher sensitivity of AI in both emphysema and non-emphysema groups compared to the results of the main analysis. Also, the sensitivity in emphysema tended to be higher for AI than for the human reader, but there were no significant differences for either the emphysema (*p* = 0.310) or the non-emphysema group (*p* = 1.000).

**Table 3:** Comparison of test performance for emphysema and non-emphysema groups between artificial intelligence (AI) and human reader (HR) excluding typical perifissural nodules and bronchovascular lymph nodes

	Sensitivity (95% CI)	PPV (95% CI)	FPs/scan	TPs	FPs	FNs	All findings
AI							
Emphysema	0.80 (0.68, 0.89)	0.73 (0.61, 0.82)	0.51	53 (61.6%)	20 (23.3%)	13 (15.1%)	86 (100%)
Non-emphysema	0.79 (0.69, 0.86)	0.81 (0.71, 0.88)	0.22	77 (66.4%)	18 (15.5%)	21 (18.1%)	116 (100%)
HR							
Emphysema	0.71 (0.59, 0.81)	0.89 (0.76, 0.95)	0.15	47 (65.3%)	6 (8.3%)	19 (26.4%)	72 (100%)
Non-emphysema	0.80 (0.70, 0.87)	0.78 (0.68, 0.85)	0.27	78 (65.0%)	22 (18.3%)	20 (16.7%)	120 (100%)

*CI* Confidence interval, *PPV* Positive predictive value, *FPs/scan* False positives per scan, *TPs* True positives, *FPs* False positives, *FNs* False negatives

Lastly, analyses on the whole dataset were performed separately for each nodule size category. This revealed differing outcomes for the 30–100 mm^3^ and 101–300 mm^3^ nodule size category (Table 4). The sensitivity of AI for lung nodule detection was higher for the larger size category compared to the smaller size category in emphysema and non-emphysema while no clear difference in sensitivity was found for the human reader across size categories. Regardless of emphysema presence, the sensitivity of AI tended to be higher than that of the reader for the 101–300 mm^3^ category, while the reader tended to show higher sensitivity for the 30–100 mm^3^ category. In the 101–300 mm^3^ category, AI had more FPs/scan than the human reader in the emphysema and non-emphysema groups (*p* = 0.010 for both). For nodular findings sized 30–100 mm^3^, the human reader had more FPs/scan than AI, in individuals without emphysema (*p* = 0.009).

**Table 4:** Comparison of results for nodule detections by artificial intelligence (AI) and human reader (HR) for emphysema and non-emphysema for different nodule volume subgroups

Ground truth by radiologists for discrepancies	Sensitivity (95% CI)	PPV (95% CI)	FPs/scan	All findings
AI 30–100 mm^3^				
Emphysema	0.59 (0.46, 0.71)	0.82 (0.67, 0.91)	0.21	71 (64.0%)
Non-emphysema	0.65 (0.55, 0.74)	0.93 (0.84, 0.98)	0.06	113 (73.4%)
AI 101–300 mm^3^				
Emphysema	0.89 (0.71, 0.97)	0.68 (0.50, 0.81)	0.31	40 (36.0%)
Non-emphysema	0.93 (0.75, 0.99)	0.67 (0.50, 0.80)	0.16	41 (26.6%)
HR 30–100 mm^3^				
Emphysema	0.79 (0.67, 0.88)	0.91 (0.79, 0.97)	0.13	68 (70.1%)
Non-emphysema	0.81 (0.72, 0.87)	0.81 (0.72, 0.88)	0.24^*^	128 (81.0%)
HR 101–300 mm^3^				
Emphysema	0.68 (0.48, 0.83)	0.95 (0.73, 1.00)	0.03^*^	29 (29.9%)
Non-emphysema	0.79 (0.59, 0.91)	0.92 (0.72, 0.99)	0.02^*^	30 (19.0%)

*CI* Confidence interval, *FPs/scan* False positives per scan, *PPV* Positive predictive value

*Significant difference between AI and HR (*p* < 0.050)

## Discussion

The results of our analyses show that the sensitivity of AI software for lung nodule detection in low-dose chest CT was similar in emphysema and non-emphysema, with higher sensitivity for larger-sized, relevant nodules (101–300 mm^3^). Similarly, the presence of emphysema did not appear to impact the sensitivity of the human reader. While overall, the sensitivity of the human reader tended to be higher than AI, this trend reversed after the exclusion of benign lymph nodes from the analysis. AI had more FPs/scan for participants with emphysema compared to the non-emphysema group, which was not present for the human reader. FPs/scan were higher for AI than the human reader in emphysema, particularly for nodule volume 101–300 mm^3^, while FPs/scan for the human reader were higher for the 30–100 mm^3^ nodules in non-emphysema.

AI’s sensitivity for nodules in which follow-up is indicated (101–300 mm^3^) was higher than for smaller-sized nodules and tended to be higher than that of the human reader in both emphysema and non-emphysema. These findings suggest that AI can assist radiologists particularly in detecting relevant nodules, some of which need follow-up (101–300 mm^3^). However, it is worth noting that this gain in sensitivity was accompanied by a higher FP rate, especially in patients with emphysema. Because a higher FPs/scan rate was observed in the emphysema group, it could be hypothesized that the underlying causative factor of the higher FP rate for AI is related to the presence of emphysema. Most FPs for AI in the presence of emphysema were identified as fibrosis/scars. One interesting finding of our study is that the reader detected more FPs/scan in the volume subgroup 30–100 mm^3^. These FPs were mostly “other” non-nodules. The presence of these FPs could be attributed to the fact that the human reader was stricter in noting a finding if it was above 100 mm^3^, as findings above 100 mm^3^ usually require follow-up, and more lenient if the finding was smaller than 100 mm^3^.

Our results show a relatively higher sensitivity of the human reader compared to AI, specifically for smaller nodules. One possible explanation is that the trained technical physician who performed the original evaluation under the supervision of the radiologist might have spent extended time per scan for detecting nodular findings, as a focus of the research study. This high sensitivity may also explain the higher FP rate for the human reader in the non-emphysema group. The human reader was very sensitive in detecting intrapulmonary lymph nodes. If we compare these results to the results in which lymph nodes (PFNs and bronchovascular lymph nodes) were excluded, the mean sensitivity of AI importantly increased in both emphysema and non-emphysema cases and tended to be better than that of the human reader in emphysema cases. These results could be explained if the AI software is optimized to detect true nodules of larger sizes and not typically benign lymph nodes, without the need for further diagnostic management [25].

Various studies have compared the performance of AI algorithms *versus* radiologists and highlighted higher sensitivity and FP rate when AI is used as a concurrent or second reader [26–29]. AI algorithms were found to have a mean number of FPs/scan ranging from 0.09 to 3.8 and a mean sensitivity (on a nodule basis) ranging from 0.54 to 0.96 [28]. In a study by Li et al. [30], it was shown that deep learning algorithms not derived only from the LIDC-IDRI dataset [31] achieved good performance in nodule detection when tested on an external dataset, with sensitivity ranging from 0.75 to 1.00. These results are in line with our study for the AI non-emphysema groups (sensitivity ranged from 0.62 to 0.78 and FPs/scan were 0.22).

One of the strengths of our study was the inclusion of study participants from a large general population cohort, not unlike a screening cohort, where AI software will be essential to enable the evaluation of a large number of CT scans. The CT scans of our participants were all acquired through a standardized acquisition protocol, with well-defined settings. Furthermore, discrepant nodules were evaluated by the expert panel without knowledge of the source of the finding (AI or human reader). Another strength is that despite the relatively small sample size, the higher FPs/scan for AI in the emphysema group was primarily associated with scars and therefore likely with the presence of emphysema itself [32]. This suggests that emphysema is indeed a factor to take into account when creating benchmark datasets for lung nodule detection.

This study has several limitations. First, the classification of emphysema was performed for most cases by one trained reader, which may have introduced some degree of subjectivity. However, since only the most extreme emphysema classifications (at least moderate emphysema *versus* no—not even trace—emphysema) were included in the study, this bias is likely negligible. Second, the size-matching of nodules was suboptimal, because the matching procedure resulted in more participants and nodules in the non-emphysema group. Also, two nodules in the emphysema group could not be matched with nodules of similar size in the non-emphysema group. A small set of subsolid nodules were also used in volume-based matching which could have resulted in selecting similar-sized, but purely solid nodules in the emphysema group. Despite these limitations, the relative distribution of nodules with size 30–300 mm^3^ was found to be similar in emphysema and non-emphysema groups after matching. Third, non-discrepant nodules by AI and human reader were not reviewed by radiologists and were considered actual nodules; these may have included some FPs. Conversely, some nodules may have been missed by both human reader and AI. In view of the purpose of this study, the consensus panel did not review all scans, only discrepancies. Fourth, the distribution of the severity of emphysema was biased toward low severity because participants were derived from a general population study. Lastly, there was no information available on the training data used to develop the algorithm. However, the decreased performance of the AI algorithm in emphysema cases most plausibly points to a lack of representation of emphysema cases in the training dataset.

Given the limitations presented above, as well as the limited number of patients with emphysema in our study, future research should focus on a larger emphysema cohort that includes also severe stages of emphysema. In a benchmark dataset that would be used to validate AI software for lung nodule detection, the inclusion of emphysema cases is crucial since we show that this can be a factor that influences its performance. Most of the published research on the performance of such algorithms has not been externally validated, and therefore, sources of bias might not have been adequately addressed [33]. It is also recommended to assess the performance of other commercial software packages in lung nodule detection in emphysema and non-emphysema cases.

In summary, the lung nodule sensitivity of AI was similar in emphysema *versus* non-emphysema groups but AI had more FPs/scan in the emphysema group. These FPs can be explained by an increased identification rate of fibrosis/scars as nodular findings. If a benchmark dataset to validate the performance of AI software in lung nodule detection is to be created, the inclusion of emphysema cases is important to evaluate the expected higher FP rate in these cases.

## Data Availability

The dataset generated and/or analyzed during the current study is not publicly available. Access to data can be requested via the Lifelines organization: https://www.lifelines.nl/researcher/how-to-apply The code that was used in this study to match nodules, extract information for the size of findings, and create images of discrepancies for the radiologists to review has been uploaded to GitHub (https://github.com/nsourlos/emphysema_experiment).

## Abbreviations

AI: Artificial intelligence
CI: Confidence interval
CT: Computed tomography
FNs: False negatives
FPs: False positives
ImaLife: Imaging in Lifelines
PFNs: Perifissural nodules
PPV: Positive predictive value
TNs: True negatives
TPs: True positives
