# Correction: Effect of emphysema on AI software and human reader performance in lung nodule detection from low-dose chest CT

**DOI:** 10.1186/s41747-024-00494-6

**Published:** 2024-08-16

**Authors:** Nikos Sourlos, GertJan Pelgrim, Hendrik Joost Wisselink, Xiaofei Yang, Gonda de Jonge, Mieneke Rook, Mathias Prokop, Grigory Sidorenkov, Marcel van Tuinen, Rozemarijn Vliegenthart, Peter M. A. van Ooijen

**Affiliations:** 1 Department of Radiology, University Medical Center of Groningen, Groningen, 9713GZ The Netherlands; 2 Department of Oral Surgery of the Medical Spectrum Twente (MST), Enschede, 7500KA The Netherlands; 3 DataScience Center in Health (DASH), University Medical Center Groningen, Groningen, 9713GZ The Netherlands; 4 Department of Epidemiology, University Medical Center Groningen, Groningen, 9713GZ The Netherlands; 5 Department of Radiology, Martini Hospital, Groningen, 9728NT The Netherlands; 6 Department of Radiation Oncology, University Medical Center Groningen, Groningen, 9713GZ The Netherlands

**Correction to:** ***European Radiology Experimental***

10.1186/s41747-024-00459-9, published online 20 May 2024

In the original article, the results section “Performance evaluation and comparison” displays two statements that the authors wish to clarify to remove ambiguity:

1. On page 6, “Sensitivity of AI was not significantly different for either the emphysema (*p* = 0.320) or the non-emphysema group (*p* = 0.090).”, should instead read: “Sensitivity was not significantly different between the emphysema and non-emphysema group for either AI (*p* = 0.80) or human reader (*p* = 0.54).”
2. On page 7, “Also, the nodule detection sensitivity in emphysema tended to be higher for AI than the human reader, but there were no significant differences for either the emphysema (*p* = 0.310) or the non-emphysema group (*p* = 1.000).” should instead read: “Also, the nodule detection sensitivity in emphysema tended to be higher for AI than the human reader, but no significant differences were found between the emphysema and the non-emphysema group for either AI (0.94) or human reader (*p* = 0.29).”

